# Comprehensive multiplatform biomarker analysis of 199 anal squamous cell carcinomas

**DOI:** 10.18632/oncotarget.6202

**Published:** 2015-10-20

**Authors:** Brandon G. Smaglo, Anteneh Tesfaye, Thorvardur R. Halfdanarson, Joshua E. Meyer, Jue Wang, Zoran Gatalica, Sandeep Reddy, David Arguello, Patrick M. Boland

**Affiliations:** ^1^ The Ruesch Center for the Cure of GI Cancers, Georgetown Lombardi Comprehensive Cancer Center, Washington, DC, USA; ^2^ Departments of Hematology/Oncology, Georgetown Lombardi Comprehensive Cancer Center, Washington, DC, USA; ^3^ Department of Medicine, Mayo Clinic, Rochester, MN, USA; ^4^ Department of Radiation Oncology, Fox Chase Cancer Center, Philadelphia, PA, USA; ^5^ Division of Oncology, University of Arizona Cancer Center, Phoenix, AZ, USA; ^6^ Department of Pathology, Caris Life Sciences, Phoenix, AZ, USA; ^7^ Department of Medicine, GI Center, Roswell Park Cancer Institute, Buffalo, NY, USA

**Keywords:** anal squamous cell carcinoma, biomarker analysis, tumor profile

## Abstract

Anal squamous cell carcinoma (ASCC) is a rare, HPV-associated malignancy typically diagnosed in early stages and definitively treated with chemoradiation. In situations where patients exhibit metastatic or recurrent disease, treatment options are severely limited. In this study, molecular alterations were identified that could be used to aid in therapeutic decisions for patients with metastatic or recurrent anal squamous cell carcinoma. Specimens from patients with this cancer were tested via a multiplatform profiling service (Caris Life Sciences, Phoenix, AZ) consisting of gene sequencing, protein expression by immunohistochemistry, and gene amplification with *in situ* hybridization. Utilizing these techniques, novel treatment strategies that could be explored were identified, including potential benefit with anti-EGFR therapies, immune checkpoint inhibitors, topoisomerase inhibitors, and taxanes. The frequency of overexpression of proteins that mark resistance to chemotherapeutic drugs, such as MRP1 (chemotherapy efflux pump), ERCC1 (resistance to platinum-based chemotherapy), and thymidylate synthase (resistance to fluoropyrimidines) were also identified, suggesting a lack of benefit. This multiplatform strategy could be explored for its potential to generate a personalized treatment selection for patients with advanced ASCC, provide a guide for future therapeutic development for this cancer, and be extended to other rare cancer types as well.

## INTRODUCTION

Anal squamous cell carcinoma (ASCC) is a rare, HPV-associated malignancy accounting for 2.5% of digestive system malignancies. In 2015, there are expected to be about 7270 new cases of anal carcinoma with 1010 deaths from the disease in the United States [1]. A review of the 1973-2000 SEER database has shown that the incidence of anal carcinoma has been increasing over the last 30 years in the general population, especially among all men [2]. Review of the National Cancer Database between 1985 and 2000 showed that on initial presentation, patients present with stages I, II, III and IV at rates of 25.3%, 51.8%, 17.1% and 5.7%, respectively [3]. The five-year overall survival rates for patients who had stages I, II, III and IV disease were 69.5%, 59%, 40.6% and 18.7 %, respectively [3].

In local and locally advanced anal squamous cell carcinoma, the standard of care for most patients is concurrent chemoradiation therapy, with a complete response rate of 80% [4]. Albeit effective, this has been the basic therapeutic paradigm for decades. Chemotherapy typically consists of a doublet of mitomycin-c (MMC) and a fluoropyrimidine administered concurrent to radiation. Flam and colleagues randomized 310 patients to receive radiation therapy (XRT) (45-50.4 Gy) along with either 5FU 1000 mg/m2 or 5FU and MMC (10mg/m2/dose for two doses). At 4 years, the 5FU/MMC arm had higher colostomy free and disease free survival rates. Surgical resection after failure of chemoradiation comes with morbid complications, but offers some remaining opportunity for cure [6, 7].

As metastatic ASCC is rare, there is limited experience to guide management. Treatment of systemic disease consists of chemotherapy administered with palliative intent, where the most accepted regimen used is cisplatin and 5FU. This use of a doublet of cisplatin and 5FU has demonstrated overall survival rates of 62.2% and 32.2% at 1 and 5 years, respectively [8]. Although there are reports on the use of different single agents and combined chemotherapy regimens in next line treatment of metastatic disease, these largely consist of single institution experiences or early phase clinical trials. A retrospective study conducted by Eng et al summarized a cohort of 76 metastatic anal cancer patients treated at a single institution. In their series, 5FU with cisplatin (PF) and carboplatin with paclitaxel (CP) were most commonly utilized as first line therapy. Although not statistically significant, this study suggested that PF has a median progression free survival of 8 months compared to only 4 months CP. The overall response rate was additionally higher with PF at 86% compared 54% with CP. Interpretation of this data is noted to be limited by an absence of prognostic information of the patients [9].

There are no approved targeted treatment options for patients with anal squamous cell carcinoma, although there are multiple anecdotal reports demonstrating the therapeutic benefit of the anti-EGFR monoclonal antibody cetuximab in the metastatic setting in chemotherapy-refractory cases [10-12]. Like HPV-associated squamous cell carcinomas arising elsewhere, anal squamous cell carcinoma has been shown to have a high frequency of expression of EGFR; whether or not its expression correlates to anti-EGFR therapeutic benefit remains an area of active investigation [11, 13]. A case series of seven patients showed that the response to cetuximab might be related to wild-type *KRAS* status, as is known to occur with its treatment of colorectal adenocarcinomas [10]. Whether or not KRAS wild-type status in ASCC is requisite for benefit from EGFR targeted therapy requires further investigation. Separately, a Danish study suggests that mutations of the *KRAS, NRAS, or BRAF* genes are rare in anal squamous cell carcinoma [14]. There are reports indicating combining cetuximab with cisplatin and 5-fu based chemoradiation in the non-metastatic setting has resulted in unacceptable toxicity [15, 16].

The lack of standard of systemic therapies for management of anal squamous cell carcinoma in the advanced setting represents an unmet need, one that is difficult to explore through a conventional clinical trial manner given the limited number of cases. Therefore, the analysis of molecular differences from available tumor specimens is an attractive source for the generation of novel therapies in the management of this cancer type. The purpose of this study was to identify novel molecular aberrations using a multiplatform approach in tumor samples of anal squamous cell carcinoma to identify and guide therapeutic treatment decisions in the metastatic setting.

## RESULTS

Using several techniques, 199 cases of anal squamous cell carcinoma were profiled for therapeutic targets or markers of drug susceptibility. Methods of evaluation included immunohistochemistry (IHC), *in situ* hybridization (ISH), and next generation gene sequencing (NGS). Samples were limited and known targets to be considered evolved over time, and thus not all samples were evaluated by all methods, nor for every target evaluable by each method.

**Figure 1:**
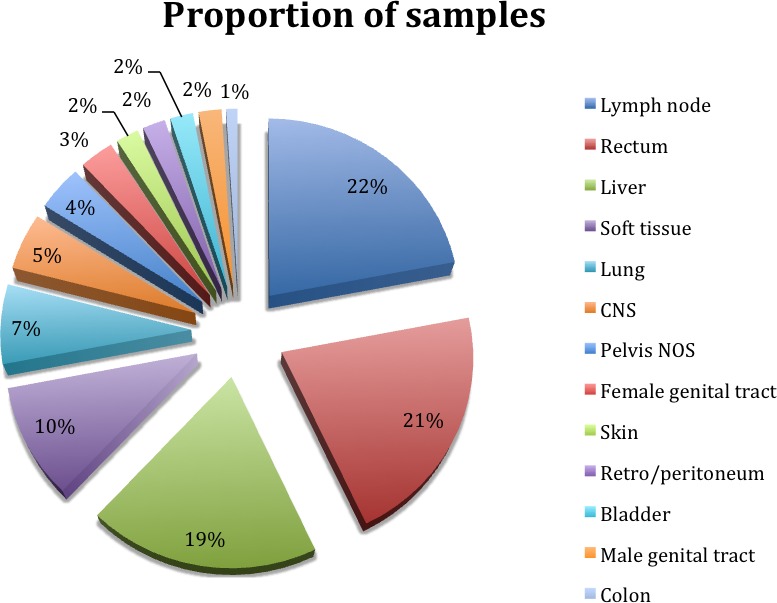
Distribution of sites of the submitted formalin fixed paraffin embedded samples of metastatic disease

Women comprised 126 (63.3%) cases while men comprised 73 (36.7%). The mean age and range of the study subjects was 58.7 years and 31-89 years, respectively. Six cases were documented as positive for HPV or HIV; status was not provided on the remaining 193 cases. The majority of tumor samples were obtained from lymph nodes (pelvis and inguinal), rectum, or liver; frequency of tumor sample location by each organ site is summarized in Figure 1. No clear relationships between demographics and the resultant tumor profiles were identified.

**Figure 2:**
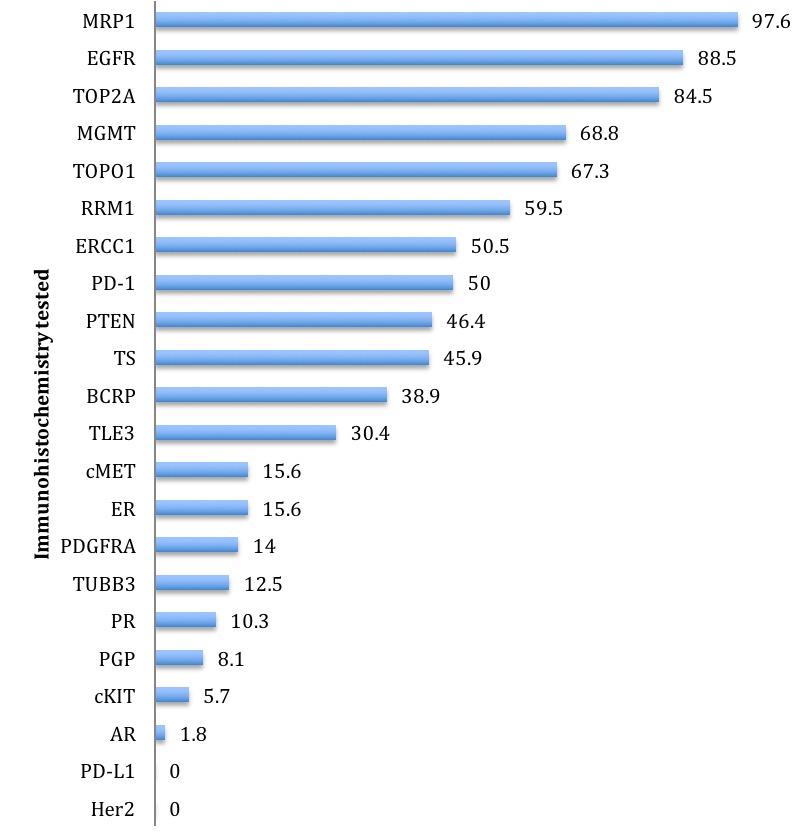
Percentages of biomarkers found to be positive by theranostic immunohistochemistry (IHC), as ranked from highest to lowest

The frequency of expression and the ratio of various protein biomarkers identified in the tumor samples by immunohistochemistry are summarized in Table 1. The frequency of expression is depicted in Figure 2, noting that sample sizes reported in the corresponding Table 1 can help clarify the true ranking of these percentages. In summarizing these results, the biomarkers expressed in any of the tumor samples evaluated by IHC that have pharmacologic targets, based upon available biomarker compendia, and markers identified that suggest a particular pharmacologic resistance are included. From the IHC results, two targets of interest in current therapy of cancer management, HER2 and PD-L1, were not identified in any of the evaluated samples. There were 180 samples evaluable for HER2 and 12 samples evaluable for PD-L1 by IHC.

**Table 1:** The distribution of protein biomarker as identified by IHC, summarized by frequency and by ratio of positive to total number tested

Biomarker by IHC	Frequency % (n positive/N tested)
MRP1*	97.6 (81/83)
EGFR	88.5 (23/26)
TOP2A	84.5 (131/155)
MGMT*	68.8 (119/173)
TOPO1	67.3 (113/168)
RRM1*	59.5 (100/168)
ERCC1*	50.5 (54/107)
PD-1	50 (6/12)
PTEN*	46.4 (83/179)
TS*	45.9 (79/172)
BCRP*	38.9 (14/36)
TLE3	30.4 (28/92)
ER	15.6 (27/173)
cMET	15.6 (14/90)
PDGFRA	14 (6/43)
TUBB3*	12.5 ((9/72)
PR	10.3 (18/174)
PGP*	8.1 (12/149)
cKIT	5.7 (5/88)
AR	1.8 (13/168)
HER2	0/180
PD-L1	0/12

* Footnote indicates biomarkers with increased resistance to a drug and/or drug class when expressed.

*In situ* hybridization was also performed to assess amplification of *ERBB2* (i.e. HER2), as well as *EGFR*. Contrary to what was observed with the IHC analysis, 2% of the 99 cases analyzed did reveal amplification of *HER2*. Additionally, amplification of *EGFR* was identified in 7.4% of tumors evaluated. Select tumor samples were evaluated by ISH for *MET TOP2A*, and *ALK*, with no genetic aberrations (i.e. amplification or rearrangement) detected. The results of these ISH analyses are summarized in Table 2.

**Table 2:** The frequency of gene amplification within anal squamous cell carcinoma as determined by *in situ* hybridization [ISH] is summarized

ISH test	Frequency % (n positive/N tested)
*EGFR*	7.4 (5/68)
*HER2*	2 (2/99)
*MET*	0 (0/69)
*TOP2A*	0 (0/18)
*ALK*	0 (0/3)

Next generation gene sequencing identified several mutations involving the PIK3CA/AKT pathway, including *PIK3CA PTEN AKT1*, and *FBXW7*. In specimens evaluated by NGS, *KRAS* was mutated in 2%. A number of gene mutations with therapeutic implications were not found in any of the samples evaluated. The frequency of the various mutation rates are summarized in Table 3a, and those mutations tested for but not identified are summarized in Table 3b.

**Table 3a:** The frequency of mutation rates within anal squamous cell carcinoma as determined by next generation sequencing (NGS) or Sanger sequencing is summarized

Gene	Platform	Frequency % (n positive/N tested)
*PIK3CA*	NGS	32.6 (28/86)
*PIK3CA*	Sanger	23.3 (7/30)
*TP53*	NGS	14.8 (8/54)
*FBXW7*	NGS	14 (8/57)
*BRCA1*	NGS	12.5 (1/8)
*BRCA2*	NGS	12.5 (1/8)
*JAK3*	NGS	5.3 (3/57)
*ERBB2*	NGS	3.6 (2/56)
*KRAS*	Sanger	2 (2/99)
*PTEN*	NGS	1.9 (1/53)
*VHL*	NGS	1.8 (1/55)
*RET*	NGS	1.8 (1/56)
*ERBB4*	NGS	1.8 (1/56)
*APC*	NGS	1.8 (1/56)
*ABL1*	NGS	1.8 (1/56)
*SMAD4*	NGS	1.8 (1/57)
*MET*	NGS	1.8 (1/57)
*AKT1*	NGS	1.8 (1/57)
*CTNNB1*	NGS	1.5 (1/68)

**Table 3b:** Genes tested for mutations with no samples testing positive, is summarized by number tested, as determined by next generation sequencing (NGS) or Sanger sequencing

Gene	Platform	N tested
*ALK*	NGS	68
*ATM*	NGS	67
*BRAF*	NGS	68
*CDH1*	NGS	68
*cKIT*	NGS	68
*CSF1R*	NGS	68
*EGFR*	NGS	68
*FGFR1*	NGS	68
*FGFR2*	NGS	68
*FLT3*	NGS	67
*GNA11*	NGS	55
*GNAQ*	NGS	39
*GNAS*	NGS	68
*HNF1A*	NGS	54
*HRAS*	NGS	54
*IDH1*	NGS	68
*JAK1*	NGS	68
*KDR*	NGS	68
*KRAS*	NGS	68
*MLH1*	NGS	68
*MPL*	NGS	68
*NOTCH1*	NGS	67
*NPM1*	NGS	68
*NRAS*	NGS	68
*PDGFRA*	NGS	68
*PTPN11*	NGS	68
*RB1*	NGS	67
*SMARCB1*	NGS	67
*SMO*	NGS	53
*STK11*	NGS	66
*BRAF*	Sanger	37
*cKIT*	Sanger	19
*EGFR*	Sanger	4
*NRAS*	Sanger	15

In 12 of the tumor samples evaluated, more than one mutation was identified in a single tumor sample by NGS. Eleven cases demonstrated dual mutations while one case demonstrated four simultaneous mutations. The composition of these various mutation combinations are summarized in Table 4.

**Table 4:** In 12 cases, more than one simultaneous gene mutation was identified in a single tumor sample by NGS. The compositions of the multi-mutations are summarized

Number of co-mutations	Number of cases	Mutation combinations
2	11	*ABL1 + ERBB4* *BRCA1 + TP53* *MET + PIK3CA* *FBXW7 + PIK3CA* *JAK3 + TP53* *PIK3CA + RET*
4	1	*APC + FBW7 + PIK3CA+ SMAD4*

## DISCUSSION

### Therapeutic implications of profiling results

To our knowledge, this is the most comprehensive molecular profiling of anal squamous cell carcinomas. The results of IHC, ISH, and sequencing provide several insights with potentially valuable clinical information, both in terms of understanding why certain standard therapies prove ineffective, as well as suggesting future areas of therapeutic exploration. Overall, 60% of tumors demonstrated some degree of dysregulation along this targetable pathway.

The overexpression of several proteins, as identified by IHC, further advances the understanding of why certain cytotoxic therapies may be ineffective in the treatment of this cancer. For example, a near-universal overexpression of multi-drug resistance-associated protein 1 (MRP1) is identified which, as discussed by Tamaki [17], leads to conventional cytotoxic therapy resistance *via* drug efflux. The overexpression of excision repair cross-complementing gene 1 (ERCC1), which has been described to confer resistance to platinum-based chemotherapy by Jiang [18] and others across various malignancies, is identified by IHC in half of the evaluated tumors. Similarly, thymidylate synthase (TS), which is identified in about 45% of the evaluated tumors, leads to fluoropyrimidine resistance, as described by Subbarayan [19]. The expression of these resistance-conferring proteins, as well as others that are indicated in Figure 2 and Table 1, advance the understanding of why advanced anal squamous cell carcinomas may be resistant to certain cytotoxic chemotherapy classes.

This profiling also suggests potential therapeutic options. EGFR was overexpressed by IHC in almost 90% of the tumors evaluated, suggesting a possible role for anti-EGFR targeting therapies such as cetuximab. This is similar to what is observed with squamous cell carcinomas of the head and neck, although EGFR expression has not universally correlated to better response to EGFR-directed therapies across all cancer types [20]. ISH analysis suggested few tumors had EGFR mutations. Moreover, only 2 tumors demonstrated mutations, which, as is the case in colorectal adenocarcinoma, has been suggested to confer resistance to anti-EGFR antibody therapy in ASCC [14]. This low mutation frequency suggests the limitation of anti-EGFR therapy associated with mutation would be rarely significant. This is supported by work previously done by Paliga [21], who found a greater than 90% rate of EGFR expression and a low rate of mutation. The use of cetuximab in the management of these cancers has already been explored by Lukan [10] with encouraging results as an alternative to standard chemotherapy, albeit in a small cohort of 7 patients. The results of this current analysis further emphasize the potential benefit of cetuximab as systemic therapy. Thus, an important conclusion of this analysis is that further formal analysis of anti-EGFR therapy is merited for anal squamous cell carcinomas.

An important additional therapeutic benefit generated by this profile is the identification of mutations that are targetable but occur with a lower frequency. Although individually occurring in smaller numbers, cumulatively, dysregulation of the PI3K/ATK/mTOR pathway was identified in 60% of the anal squamous cell carcinomas evaluated, consistent with what is being reported. Targets within this pathway have been previously considered. Patel [22] has described AKT activation in a retrospective analysis of 128 patient tumors, and concluded that AKT has an important component of anal squamous cell carcinoma growth, thus suggesting it as a potential therapeutic target in this disease. In a transgenic mouse model, Sun [23] has evaluated the role that mTOR plays in anal carcinogenesis, finding that mTOR knockout led to a delay in carcinoma onset, and thus suggesting this as a potential therapeutic target, too.

This profiling of ASCC also identified several other therapeutic targets that could warrant investigation, based upon their action-ability in other cancer types. Almost a third of tumors evaluated were non-expressers of methylguanine-DNA-methyltransferase (MGMT) by IHC, suggesting a possible susceptibility to temozolomide. Of the 12 tumor samples evaluated for PD-1 expression by IHC, 50% were expressive, and this could suggest a possible role for an immunotherapeutic agent such as nivolumab or pembrolizumab. Of note, none of 24 tumor samples that were evaluated for PD-L1 demonstrated expression. No evaluations of the use of anti-PD-1 therapy in the management of anal squamous cell carcinoma are currently reported in the literature. However, while not therapeutically evaluated, a retrospective study of PD-L1 positivity was associated with a non-statistically significant trend towards a poorer RFS in ASCC, consistent with what has been observed with other cancer types [24]. Finally, there were additionally a small subset of tumors found to have HER2 amplification by ISH, and when identified, these tumors could potentially be candidates for therapy with HER2-targeting agents.

The targets occurring in smaller subsets of patients’ tumors illustrate a key difference in appropriate investigational strategy, as compared to the case with cetuximab above. Since these targets are identified in the minority of tumors, it would not be appropriate for the agents that target them to be explored in an all-comer cohort of anal squamous cell carcinomas. Rather, if tumor profiling could be conducted in advance of therapy, those specific patients likely to benefit from specific targeting agents could be selected. It is precisely in these cases that a tumor profiling platform such as the one described here can be applied to a clinical setting. There are no current studies reported in the literature that investigate the use of therapies against these targets in the management of anal squamous cell carcinoma.

### HPV associated cancers

In the larger context of cancer management, addressing anal squamous cell carcinoma has drawn increasingly strong parallels to other human papilloma virus (HPV)-related malignancies. As reviewed by Tommasino [25], the HPV subtypes etiologic for cervical cancers (HPV 16 and 18) have since been linked to anal, penile, vulvo-vaginal, and head and neck squamous cell cancers, and Forman [26] reports that up to 90% of anal squamous cell carcinomas are linked to HPV-infection. Ghosn [27] notes that there is an increasing incidence of anal cancers currently, associated with the risk of HPV transmission, particularly in the subpopulation of men who have sex with men.

As parallels from anal squamous cell carcinoma to other HPV-related cancers develop, similarities in management strategies are emerging, in terms of screening and prevention. Already, as reported by Palefsky [28], HPV vaccination reduces the occurrence of anal squamous cell cancer in the at risk population of men who have sex with men. While there is not yet a recommended route to screen for anal cancer, as reviewed by Gami [29], screening methods paralleling similar cancers, such as swabs with Papanicolaou, are being explored. Given these similarities between tumor management, the development of treatment strategies across these tumor types is a natural extension of investigation. The information garnered from this current profile of anal squamous cell carcinoma could be further augmented through the parallel profiling of these other cancers. An important consideration would be, beyond just the individual tumor profiles, the parallels or differences between these cancers’ profiles, and the immediate extension of these to parallels of drug selection. This may prove an invaluable expedition of treatment development, through the extrapolation of development of one drug from one cancer to its cousin diseases.

While less common, those ASCC that arise in the absence of HPV have been associated to mutation, and seem to be treatment resistant in comparison to their HPV-associated counterparts [30]. These HPV-negative ASCC are also ideal candidates for novel treatment strategies, such as what can be explored with tumor profiling. Of note, p16 was not included in the IHC panel, even though it may have a stronger concordance with HPV-positivity. This was excluded because the developed panel was intended to be a tumor agnostic panel, and p16, when identified, would lack a predictive utility. However, p16 inclusion in future IHC panels is certainly worth strong consideration.

### Strengths and limitations

This initial retrospective analysis has many limitations. The dearth of clinicopathologic information limited our ability to further identify groups of anal SCC patients who may derive more benefit from certain chemotherapeutic or targeted therapies. Without this information, we were unable to stratify between patients with HPV *versus* those without HPV. Also, identification of those patients with HIV would have provided further insights. Furthermore, given the lack of clinical outcomes this analysis does not allow further analysis into whether this approach ultimately benefits patients with ASCC. A prospective trial, then, would be needed to validate the findings. Regardless, the importance of a multiplatform approach to the development of novel treatments for late-stage anal squamous cell carcinoma cannot be overemphasized. By linking therapies to tumor biology, a rationale explaining why therapies succeed or fail might be ascertained. This same knowledge could guide future patient treatments, leading to novel options that might not otherwise be considered. It should also serve as the basis for hypothesis driven clinical trials. As noted with cases including MRP1, ERCC1, TS, and others, this approach offers confirmation as to why certain therapies are ineffective.

The findings of a multiplatform approach such as this are limited therapeutically to those targets that have available treatments. In cases where therapies are not available for identified targets, profiling such as this could be useful to identifying novel targets for therapeutic development. Additionally, this approach is limited to the targets investigated and does not suggest as-yet-unknown targets that might otherwise be explored and exploited. Future trials should evaluate other biomarkers and pathways besides PIK3CA/AKT/mTOR that may be worth targeting at a later date.

## CONCLUSIONS

This profile of ASCC already has revealed a number of intriguing findings that offer insights and inroads for further therapeutic investigation. These include possible explanations of tumor resistance mechanisms to certain cytotoxic chemotherapy. EGFR expression and low rates of and mutations support further investigation of anti-EGFR therapies for this cancer type, noting that expression of EGFR has not been universally correlated to therapeutic effect of targeting across all cancer types. A number of therapies could be explored through the possibility of targeting the PI3K/ATK/mTOR pathway. The results presented here are largely in agreement with smaller, similar studies, such as the FISH analysis performed by Martin and colleagues in 2014 [31]. As these and other studies develop, the expanded body of knowledge that they provide can refine these profiling techniques in order to identify reliable marker of targets for future therapeutic development.

Currently, the options for treatment of advanced or metastatic anal squamous cell carcinoma are limited. Because advanced stages of this cancer remain uncommon, few opportunities for investigating treatment regimens in large cohorts of patients exist. Therefore, novel strategies for determining the best therapies for the treatment of these patients are required. The multiplatform analysis presented here could provide this tool.

## MATERIALS AND METHODS

One-hundred and ninety-nine anal squamous cell carcinoma specimens were tested by the Caris Life Sciences multiplatform profiling service. These specimens were referred for testing by a treating physician, and thus the scientists and clinicians at Caris evaluated only the tumors and not the actual patients. Limited demographic and clinical information was available about the patients whose tumors were analyzed, which included age and gender, and organ from which the evaluated tumor specimen was assessed.

Specimens were submitted in the form of formalin fixed paraffin embedded (FFPE) samples. A multiplatform profiling service was conducted on these samples to assess protein expression and gene aberrations. Protein expression was performed using immunohistochemistry [IHC]. Gene amplification was assessed using chromogenic *in situ* hybridization [CISH] or fluorescent *in situ* hybridization [FISH]. Gene sequencing was performed using Sanger or next generation sequencing [NGS].

### Immunohistochemistry

Immunohistochemistry analysis was performed on formalin-fixed, paraffin-embedded (FFPE) tumor samples using commercially available detection kits and automated staining techniques (Benchmark XT, Ventana, Tucson, AZ; and AutostainerLink 48, Dako, Carpinteria, CA). Antibodies for the following were utilized: androgen receptor (AR), multiple resistance protein 1 (MRP1), topoisomerases 1 and 2A (TOPO1, TOPO2A) (Leica Biosystems, Buffalo Grove, IL); estrogen receptor (ER), progesterone receptor (PR), MET proto-oncogene receptor tyrosine kinase (cMet), human epidermal growth factor receptor 2 (HER2), programmed death - one (PD-1), and programmed death ligand (PD-L1) [Ventana, Tucson, AZ]; tyrosine protein c-Kit receptor kinase (c-Kit), phosphatase and tensin homolog (PTEN) [Dako], epidermal growth factor receptor (EGFR) [Life Technologies, Carlsbad, CA]; O(6)-methylguanine-methyltransferase (MGMT), P-glycoprotein (PGP), thymidylate synthase (TS) [Invitrogen, Grand Island, NY]; DNA excision repair cross-complementing one protein (ERCC1) [ABCAM, Cambridge, MA]; breast cancer resistance protein (BCRP), transducin-like enhancer of split 3 (TLE3) [Santa Cruz, Santa Cruz, CA]; platelet-derived growth factor alpha (PDGFRA) [Thermo Scientific, Waltham, MA]; ribonucleotide reductase M1 (RRM1) [Protein Tech, Chicago, IL]; and tubulin beta-3 chain (TUBB3) [Covance, Madison, WI]. Scoring system and cutoffs for all antibodies are provided in Supplemental Table 1.

### *In situ* hybridization

Fluorescent *in situ* hybridization (FISH) evaluated for amplification of the ([chromosome 17 centromere] probe), (probe), (probe; Abbott Molecular/Vysis, Abbott Park, IL), and (probe) genes. The ratio > 2.2 was considered amplified (based on guidelines from the College of American Pathology [CAP]/ASCO [American Society of Clinical Oncology], 2007). amplification was defined as > = 2, or > = 15 EGFR copies per cell in > = 10% of analyzed cells. was considered amplified if > = 5 copies were detected, while amplification was defined as TOP2A/CEP17 ratio > = 2.0. ALK was also assessed using FISH in a few patients for gene rearrangement (Abbott Molecular/Vysis, Abbott Park, IL).

*HER2* and *MET* status were also evaluated using chromogenic *in situ* hybridization (INFORM *HER2* Dual ISH DNA Probe Cocktail; commercially available *MET* and chromosome 7 DIG probe; Ventana, Tucson, AZ). The same scoring system was applied as for FISH.

### Mutational analysis

Next-Generation Sequencing. Direct sequence analysis was performed on genomic DNA isolated from formalin-fixed paraffin-embedded tumor samples using the Illumina MiSeq platform (La Jolla, CA). Specific regions of 47 genes of the genome were amplified using the Illumina TruSeq Amplicon Cancer Hotspot panel. Sequencing depth average was 1500X.

Sanger Sequencing. Mutational analysis using Sanger sequencing involved selected regions of *KIT* and *PIK3CA* and was performed using M13-linked polymerase chain reaction (PCR) primers designed to amplify targeted sequences. PCR products were bi-directionally sequenced using the BigDye Terminator v1.1 chemistry (Applied Biosystems, Grand Island, NY), and analyzed using the 3730 DNA Analyzer (Applied Biosystems). Sequence traces were analyzed using Mutation Surveyor software v3.25 (Soft Genetics, San Francisco, CA).

### Statistical methods

The frequency of the various protein expression, and gene sequence and amplification data was compared across the tumor samples that were analyzed, to assess for frequency and trends, in order to assess for possible therapeutic targets. These results were also compared to the available demographic patient data. Data analysis was performed using SPSS 20 (IBM, Armonk, NY) Statistical software and JMPv10.0 (SAS Institute Inc, Cary, NC).

## SUPPLEMENTARY MATERIAL TABLE


