# Supplementary material for: Comprehensive multiplatform biomarker analysis of 199 anal squamous cell carcinomas
Source: Oncotarget. 2015 Oct 20;6(41):43594–604. doi: 10.18632/oncotarget.6202 (PMC4791253; doi:10.18632/oncotarget.6202)
Supplement: Supplementary file 1 [file oncotarget-06-43594-s001.pdf]

Comprehensive multiplatform biomarker analysis of 199 anal squamous cell carcinomas

Supplementary Material

Supplemental Table 1.

Biomarker(s)	Threshold for positivity/expression (based on staining intensity and percent of staining cells)
Androgen Receptor, BCRP, ER, PR, EGFR, MRP1, Pgp, TOP2A, TS	>=1+ in 10% or more
cKIT, PDGFRA, TLE3, TOPO1, TUBB3	>=2+ in 30% or more
cMET	>=2+ in 50% or more
ERCC1	2+ in 50% or more; 3+ in 10% or more
HER2	3+ in 10% or more
MGMT	>=1+ in 35% or more
PD-1	>5 tumor infiltrating lymphocyte (TIL) count per high-powered field (HPF)
PD-L1	>=2+ in 5% or more
PTEN	>=1+ in 50% or more
RRM1	>=2+ in 50% or more
